# A snapshot of the nutritional status of Crohn’s disease among adolescents in Brazil: a prospective cross-sectional study

**DOI:** 10.1186/s12876-015-0403-2

**Published:** 2015-12-08

**Authors:** Camila Ortiz Prospero Cavalcante Costa, Flair José Carrilho, Valeria Sutti Nunes, Aytan Miranda Sipahi, Maraci Rodrigues

**Affiliations:** 1 Department of Gastroenterology, University of Sao Paulo School of Medicine Hospital das Clínicas, Av. Dr. Eneas de Carvalho Aguiar 255, 05403-000 Sao Paulo, Brazil; 2 Lipids Laboratory (LIM-10), Endocrinology and Metabolism Division of University of Sao Paulo School of Medicine Hospital das Clinicas, Av.Dr.Eneas de Carvalho Aguiar 255, 05403-000 Sao Paulo, Brazil

**Keywords:** Crohn’s disease, Adolescence, Nutritional assessment

## Abstract

**Background:**

The relationship between nutrition and Crohn’s disease (CD) is complex and involves several therapeutic possibilities including: nutrition treatment for malnourished patients, optimization of growth and development, prevention of osteoporosis, first-line therapy for active disease, and maintenance of disease remission. In children and adolescents with CD, malnutrition is a common problem that adversely affects the prognosis. In at-risk adolescent CD patients, it is important to assess body composition, food intake, energy expenditure, nutrient balance and serum levels of nutrients before planning interventions for this population. The aim of this study was to provide a snapshot of the nutritional status of adolescents with CD in Brazil.

**Methods:**

We prospectively selected 22 patients with mildly to moderately active CD, 29 patients with inactive CD and 35 controls (first-degree relatives of and in the same age bracket as the CD patients). The age range of participants was between 13.2 and 19.4 years old. We collected anthropometric data including weight, height, and body mass index (BMI), which were expressed as Z scores: weight-for-age, height-for-age and BMI-for-age, respectively, as well as using bioimpedance to determine body composition and assessing the Tanner stage. We also assessed macronutrients and micronutrients (serum levels and dietary intake of both). We used the chi-square test to determine whether any of the studied variables were associated with inactive or active CD. The level of significance was set at 5 % (*p* < 0.05). We have written informed parental consent for participation for any minors and written informed consent for any participants that were adults.

**Results:**

The mean values for lean body mass, Tanner stage, height-for-age Z score and BMI-for-age Z score were lower in the active CD group than in the inactive CD and control groups (*p* < 0.05 for both). Compared with the controls, the CD patients showed significant differences in terms of the quality of dietary intake (particularly in caloric intake, dietary protein intake, dietary fiber intake, and micronutrient intake), which were reflected in the serum levels of nutrients, mainly vitamins A and E (*p* < 0.05).

**Conclusions:**

Adolescents with CD (including those with mildly to moderately active or inactive disease) have a nutritional risk, which makes it important to conduct nutritional assessments in such patients.

## Background

In approximately 25–30 % of all patients with CD, the onset of the disease occurs before 20 years of age. Because the peak onset of pediatric inflammatory bowel disease (IBD) occurs in late adolescence, this event may turn out to be a potent influence on puberty and growth development [1–3].

In this setting, the most specific complication of pediatric CD is growth deficit, which is caused by a combination of inadequate caloric intake, increased loss of calories and persistent active inflammation of the intestinal mucosa [4]. In such patients, excessive weight loss and malnutrition result in abnormal anthropometric measurements. Therefore, in patients with IBD (principally in those with CD), body mass indices (BMIs) and weights are below normal when compared with reference values or with the values reported for healthy controls [5, 6]. Particularly at this age, micronutrient deficiencies can influence the progression and clinical outcome of IBD, affecting the immune and antioxidant defense systems, as well as tissue repair, growth, and bone mineralization [7].

The presence of nutritional changes in adolescents with CD has not always been adequately investigated or given sufficient attention by physicians. Nutritional changes in such patients are directly influenced by the society in which they live and the treatment given. Therefore, data from other countries cannot be extrapolated to Brazil. This, together with the lack of studies examining this issue in the country, motivated us to conduct the present study.

## Methods

The study population consisted of patients with a confirmed diagnosis of CD [1] undergoing regular treatment at the Clinical Gastroenterology Outpatient Clinic of the University of São Paulo School of Medicine *Hospital das Clínicas*, located in the city of São Paulo, Brazil, between January 2007 and March 2011. The controls were selected among individuals who were first-degree relatives of the CD patients and were in the same age bracket as the latter. The age range of participants was between 13.2 and 19.4 years old. We excluded individuals with intestinal diseases or other diseases affecting the nutritional status. First-degree relatives were used as controls because their cultural and environmental influences were the same as those of their CD counterparts; that is, they had the same dietary habits and lived in the same city. In addition, adherence to the study protocol was higher because the controls were relatives of the CD patients. For the CD and control groups, the exclusion criteria were as follows: diagnosis of infection, cancer, severe psychiatric disorder, hypothyroidism, hyperthyroidism, corticosteroid usage in the last two months and pregnancy.

In order to classify CD in terms of its characteristics and severity, we used the Paris classification (a pediatric modification of the Montreal classification of IBD) [8] and the Pediatric Crohn’s Disease Activity Index [9].

The Research Ethics Committee of the University of Sao Paulo School of Medicine *Hospital das Clínicas*, in the city of Sao Paulo, Brazil, approved the research project.

We have written informed parental consent for participation for any minors and written informed consent for any participants that were adults.

### Anthropometry

Anthropometric variables, such as weight, height, and BMI, as well as bioimpedance, were determined in accordance with standardized methods [10, 11]. Weight and BMI were expressed as Z scores using the World Health Organization (WHO) program AnthroPlus, version 1.0.4 (http://www.who.int/growthref/tools/en/), and sexual maturation was classified according to the Tanner stage [12], as well as being ranked as pubertal or prepubertal according to the recommendations of the WHO [11].

In the bioimpedance analysis, we used a tetrapolar whole-body analyzer (BIA 310; Biodynamics Corporation, Seattle, WA, USA). According to Woodrow [13], the use of bioimpedance analysis for assessing body composition is a rapid, noninvasive and relatively inexpensive method for estimating the amount of body fat, as well as being a portable and easily applicable method.

### Dietary intake

Dietary intake was assessed by means of a 7-day food diary [14]. The food diary covered the 7 days that preceded the medical consultation, was filled out by patients themselves or their legal guardians, and was reviewed upon delivery in order to minimize procedural errors.

The quantitative variables studied were total energy intake, consumption of macronutrients (carbohydrates, lipids and proteins), and consumption of fiber and micronutrients (vitamins A, B12, C, D, E, folic acid, calcium, iron, potassium, magnesium, sodium and zinc). The calculations were made with the software DietPro, version 5.7i (A. S. Sistemas, Viçosa, Brazil).

To determine the basal metabolic rate for each adolescent, we used the Harris-Benedict equation [15], corrected for gender, age, weight and coefficient of degree of physical activity during the week, deemed as minimal effort for all patients studied. Considering 100 % of dietary reference intakes (DRIs) [16], we ranked total energy intake and micronutrient consumption as below or above the recommended levels. Macronutrient intake was analyzed according to the recommendations of the WHO and the United Nations Food and Agriculture Organization, thus categorized as below the lower limit, above the upper limit or within the recommended range [17].

### Biochemical parameters

Blood samples were collected for analysis of serum levels of the following: vitamins A, B12, and E, as well as 25-hydroxyvitamin D, folic acid, iron, ionized calcium, zinc, magnesium, potassium, copper, albumin, hemoglobin, cholesterol and triglycerides. The values obtained were analyzed against the reference values adopted by the Central Laboratory of the University of São Paulo School of Medicine *Hospital das Clínicas*.

### Statistical analysis

We used the chi-square test in order to investigate possible associations between the studied variables and the three groups (inactive CD, active CD, and control). The level of significance was set at 5 % (*p* < 0.05). The food consumption data were processed with the DietPro software, after which they were analyzed with the programs Microsoft Excel, Epi Info, version 6.04, and the Statistical Package for the Social Sciences, version 13.0 (SPSS Inc., Chicago, IL, USA).

## Results

The present study included 86 individuals: 22 in the active CD group (patients with mildly to moderately active CD), 29 in the inactive CD group, and 35 in the control group. The mean (SD) PCDAI scores (0–100 scale) for patients with inactive, mild, and moderate disease were 7.0 (7.1), 18.3 (10.8), 26.2 (11.4), respectively. The demographic characteristics of the study sample are summarized in Table 1. The clinical characteristics, location and behavior of the CD are summarized in Table 2. Patients with inactive CD showed an increase of the median duration of the disease, although the difference was not significant. In terms of the location affected, simultaneous involvement of the ileum and colon was most common, followed by isolated involvement of the ileum and isolated involvement of the colon. The most common types of CD were the non-stricturing non-penetrating type and the penetrating type.

**Table 1:** Sociodemographic characteristics of Crohn’s disease patients and controls

Characteristic	Active CD (Group I)	Inactive CD (Group II)	Control (Group III)	G1	G2	G3	
	(*n* = 22)	(*n* = 29)	(*n* = 35)				
Age (years), mean ± SD	16.4 ± 2.5	17.3 ± 2.19	15.5 ± 2.31	NS	NS	NS	
Gender							
Male, n (%)	12 (54.5)	16 (55.2)	22 (62.8)	NS	NS	NS	
Female, *n* (%)	10 (45.5)	13 (44.8)	13 (37.2)	NS	NS	NS	
Race							
White, *n* (%)	20 (90.9)	26 (89.6)	32 (91.3)	NS	NS	NS	
Non-White, *n* (%)	2 (9.1)	3 (10.4)	3 (8.7)	NS	NS	NS	

*CD* Crohn’s disease, *SD*: standard deviation, *G1* group I vs. group II, *G2* group I vs. group III, G3 group II vs. group III, NS not significant

**Table 2:** Clinical characteristics, location, and behavior of the disease in patients with active and inactive Crohn’s disease, based on Levine et al. [8]

	Active CD	Inactive CD	G1
Mean time of disease duration (years) ± SD	1.14 ± 1.06	2.83 ± 3.34	NS
Mean age of onset of symptoms (years) ± SD	14.4 ± 3.7	14.1 ± 3.7	NS
Mean age at diagnosis (years) ± SD	15.6 ± 2.38	14.5 ± 3.66	NS
Mean PCDAI ± SD	18.3 ± 10.8 26.2 ± 11.4	7.0 ± 7.1	
Location, *n* (%)			
L1(distal 1/3 ileum/limited cecal disease)	8 (36)	11 (38)	NS
L2(colonic)	3 (14)	7 (24)	NS
L3(ileocolonic)	11 (50)	11 (38)	NS
L4 a (upper disease proximal to ligament of Treitz)	0	0	
L4b (upper disease distal to ligament of Treitz and proximal to distal 1/3 ileum	0	0	
Behavior, *n* (%)			
B1: non-stricturing non-penetrating	16 (73)	25 (86)	NS
B2: structuring	2 (9)	0	NS
B3: penetrating	4 (18)	4 (14)	NS
B2B3: both penetrating and stricturing disease, either at the same or different times	0	0	
p: perianal disease modifier	2 (9)	0	NS

*CD* Crohn’s disease, *SD* standard deviation, *G1* group I vs. group II

As can be seen in Table 3, the mean Z scores for height-for-age and BMI-for-age were higher in the active CD group than in the inactive CD group, as well as being higher in the inactive CD group than in the control group; the differences among the groups were significant. When compared individually, 2 (9.1 %) of the patients with active CD and 1 (3.4 %) of those with inactive CD had short stature (height-for-age Z score < − 2). In addition, 7 (31.8 %) of the patients with active CD and 3 (10.3 %) of those with inactive CD were malnourished (BMI-for-age Z score < − 2). The deficit of lean body mass in relation to the fat compartment was more pronounced in the active CD group than in the inactive CD and control groups. It is of note that some (4.2 %) of the CD patients had a BMI-for-age Z score > 2 standard deviations, which characterized them as overweight.

**Table 3:** Anthropometric evaluation of active and inactive Crohn’s disease patients and controls

Variable	Active CD	Inactive CD	Control	G1	G2	G3
	(Group I)	(Group II)	(Group III)			
	(*n* = 22)	(*n* = 29)	(*n* = 35)			
Z score						
Ha, mean ± SD	−0.41 ± 0.90	−0.15 ± 0.92	0.24 ± 0.99	*	*	*
BMIa, mean ± SD	−1.29 ± 1.64	0.01 ± 1.18	0.37 ± 1.18	*	*	*
Bioimpedance analysis						
BF (%), mean ± SD	23 ± 0.08	21 ± 0.05	24 ± 0.04	NS	NS	NS
LBM (%), mean ± SD	74 ± 0.08	79 ± 0.05	76 ± 0.04	*	*	NS
Tanner staging						
Delayed puberty, n (%)	5 (22.7)	2 (6.9)	-	*	*	NS

*CD* Crohn’s disease, *G1* group I vs. group II, *G2* group I vs. group III, *G3* group II vs. group III, *Ha* height-for-age, *BMIa* body mass index-for-age, *BF* body fat, *LBM* lean body mass, *NS* not significant; *p < 0.05

Regarding the Tanner stage of sexual maturation, delayed puberty was observed in 22.7 % of the active CD group patients, compared with only 6.9 % of those in the inactive CD group; the difference between the two groups was significant. None of the individuals in the control group presented delayed puberty.

The patients that made the greatest use of azathioprine were those with active disease (95.4 %). At the time of this study, none of the patients were using prednisone. Antibiotics (metronidazole and ciprofloxacin) were used by approximately one-third of the active CD group patients and half of the inactive CD group patients. Approximately 23 % of the patients with active CD were using infliximab (Table 4).

**Table 4:** Distribution of the patients (%) with active or inactive Crohn’s disease according to the drugs that were taken before and during the study

Drug	Before the study		G1	During the study		G1
	Active CD	Inactive CD		Active CD	Inactive CD	
	*n* (%)	*n* (%)		*n* (%)	*n* (%)	
	(Group I)	(Group II)		(Group I)	(Group II)	
Azathioprine	22(100)	25(86.2)	NS	21(95.4)	18(62.1)	*
Sulfasalazine	3(13.6)	3(10.3)	NS	2(9.1)	1(3.5)	NS
Mesalazine	20(90.9)	15(51.7)	*	10(45.4)	16(55.2)	NS
Ciprofloxacin	12(54.5)	16(55.2)	NS	3(13.6)	12(41)	*
Metronidazole	10(45.4)	11(37.9)	NS	4(18.2)	2(6.9)	NS
Infliximab	8(36.3)	6(20.7)	NS	5(23.1)	5(17.2)	NS

*CD* Crohn’s disease, *G1* group I vs. group II, *NS* not significant; *p < 0.05

**Fig. 1:**
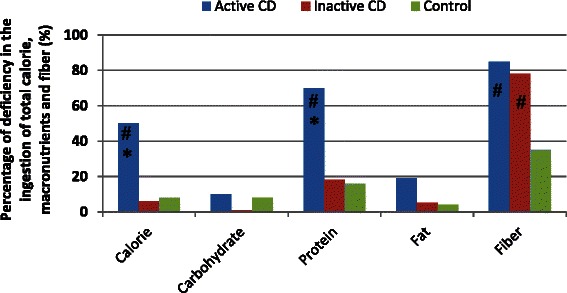
Deficiency^a^ in the ingestion of total calories, macronutrients and fiber in patients with active DC, patients with inactive DC and control individuals. ^a^ Percentange of deficiency is based on the recommendations of the World Health Organization and the United Nations Food and Agriculture Organization [17]. *p < 0.05 vs. inactive CD group; # *p* < 0.05 vs. control group

Total energy intake was lower than the DRIs in 50 % of the adolescents with active CD, in 3.5 % of those with inactive CD, and in 5.7 % of those in the control group (Fig. 1). Macronutrient intake was found to be below the DRIs in 9.1 % of the patients with active CD and in 5.7 % of the controls; no decrease in carbohydrate intake was observed in the patients with inactive CD. Protein intake was found to be below the DRIs in all three groups, being far lower in the active CD group than in the inactive CD and control group (68.2 % vs. 17.2 % and 14.3 % below the DRIs, respectively). Lipid intake was also found to be below the DRIs, principally in those with active CD (18.2 % below the DRIs). Dietary fiber intake was low in all groups, being particularly low in the two groups of patients with CD (Fig. 1).

**Fig. 2:**
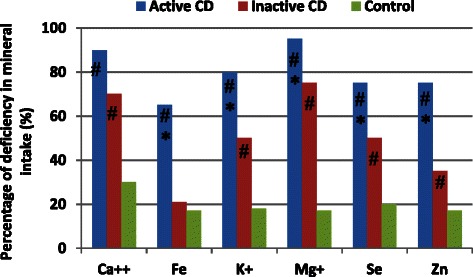
Percentage of deficiency^a^ in mineral intake in the patients with active DC, patients with inactive DC and control individuals. ^a^Percentange of deficiency is based on dietary reference intakes [16]. *p < 0.05 vs. inactive CD group; # *p* < 0.05 vs. control group

Figure 2 shows the percentage of individuals with a mineral intake below the recommended level. Dietary calcium, iron, potassium, magnesium, selenium, and zinc intake was lowest in the active CD group, followed by the inactive CD and control group; the differences among the three groups were statistically significant (*p* < 0.05 for all; Fig. 2).

**Fig. 3:**
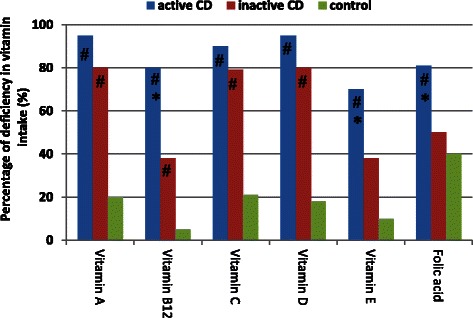
Percentage of deficiency^a^ in vitamin intake in the patients with active DC, patients with inactive DC and control individuals. ^a^Percentage of deficiency is based on dietary reference intakes [16]. *p < 0.05 vs. inactive CD group; # *p* < 0.05 vs. control group

Vitamin intake was lower than recommended in at least 75 % of the patients with active CD (Fig. 3). Vitamin intake was lower in the active CD group than in the inactive CD group, as well as being lower in the CD groups than in the control group; the differences were statistically significant (*p* < 0.05).

In comparison with the controls, the patients with CD were deficient in serum ferritin and serum albumin. In addition, serum albumin levels were lowest in the patients in remission; the difference between that group of patients and the remaining two groups showed a trend toward statistical significance (*p* < 0.05). Serum ferritin levels were also lower in the patients in remission with the difference showing a tendency toward statistical significance (*p* < 0.05 vs. the other groups).

**Fig. 4:**
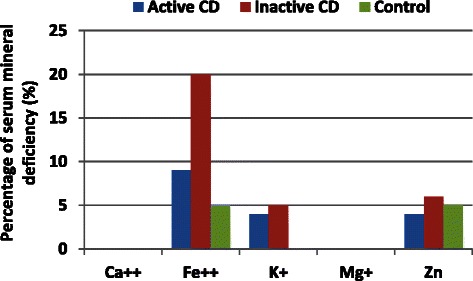
Percentage of serum mineral deficiency in the patients with active DC, patients with inactive DC and control individuals

Although calcium and magnesium intake was low, in comparison with the DRIs, in all three groups, none of the groups showed serum calcium deficiency or serum magnesium deficiency (Fig. 4). In the present study, the most common dietary mineral deficiency was serum iron deficiency (*p* < 0.05), being more pronounced in the inactive CD group than in the active CD group (in contrast to what was observed for the remaining minerals).

**Fig. 5:**
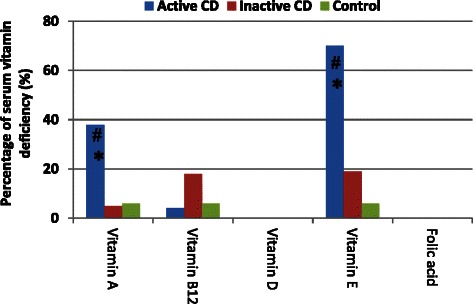
Percentage of serum vitamin deficiency in the patients with activity CD, inactive CD and control group. *p < 0.05 vs. inactive CD group; # *p* < 0.05 vs. control group

Serum levels of vitamin A and vitamin E were lower in the active CD group than in the remaining two groups (*p* < 0.05). In contrast, the rate of vitamin B12 deficiency was highest in the inactive CD group, although the differences among the groups were not significant (*p* > 0.05). None of the groups showed serum vitamin D deficiency or serum folic acid deficiency (Fig. 5).

We did not find significant differences (*p* > 0.05) for the cholesterol and triglycerides levels respectively between the inactive CD (145.76 ± 3.65 mg/dl; 94.24 ± 7.47 mg/dl), active CD (148.27 ± 2.12 mg/dl; 93.64 ± 5.58 mg/dl) and control group (147.60 ± 3.03 mg/dl; 92.23 ± 6.7 mg/dl). For instance, the hemoglobin results were lowest in the inactive and active CD group (11.86 ± 0.35 g/dl; 11.87 ± 0.39 g/dl) compared to the control group (12.15 ± 0.27 g/dl), but not significantly different.

The CD patients investigated in the present study showed significant changes in body composition and in the quality of dietary intake (particularly in caloric intake, dietary protein intake, dietary fiber intake, and micronutrient intake), which were reflected in the serum levels of nutrients, principally vitamins A and E.

## Discussion

In the present study, we provided a snapshot of the nutritional status of CD adolescents in Brazil using instruments and methods that are available at any health facility where such patients might be followed. We found that BMI-for-age and height-for-age were significantly lower in the patients with CD than in the controls, a finding that was consistent with those of Burnham et al. [18], who studied 104 children and young adults with CD evaluated an average of 4 years after the disease had been diagnosed (as was the case in the present study).

In a similar study, Thayu et al. [19] investigated a sample of 78 children and adolescents with CD and found significant changes in growth, pubertal development, and body composition (in particular, lean body mass loss) at diagnosis in comparison with the control group, although they found no differences between the genders. The deficit of lean body mass persisted throughout the follow-up period.

In the present study, caloric intake was found to be lower than recommended in 50 % of the patients with active CD, in 3.5 % of those with inactive CD, and in 5.7 % of the controls. According to Thomas et al. [20], during disease exacerbation, caloric intake can decrease by 20 % from the recommended intake and the energy deficit can reach 400 kcal per day. Those authors showed that the mean intake of all micronutrients except vitamin B12 was below the DRIs. Likewise, in the present study, the prevalence of micronutrient deficiency was higher among the patients with CD than among those in the control group. In addition, micronutrient intake was lower in the patients with active CD than in those with inactive CD.

According to Green et al. [21], dietary intake deficiencies are due to major changes in dietary habits occurring after the diagnosis of CD. The authors observed qualitative changes in the dietary habits of pediatric CD patients, who primarily avoided milk and dairy products, as well as fruits and vegetables (because of their high fiber content), mainly for fear of triggering an exacerbation.

Approximately 30 % of the CD patients in our study showed serum iron deficiency, a finding that was consistent with those of at least two studies [22, 23]. What surprised us was the fact that the prevalence of iron deficiency was higher in the patients with inactive CD than in those with active CD. One possible explanation is that many of the patients with active CD were receiving iron supplements and the hemoglobin was not statistically different between these group. In this regard, the lower iron level observed in the inactive CD group could be explained with a longer duration of the disease in comparison with the active group, although the values were not statistically different.

We also found that patients with inactive and active CD were deficient in serum albumin in comparison with the controls, and were lowest in the patients in remission, probably because the parameter used was only PCDAI and no calprotectin or endoscopy parameter was used. In contrast, El-Matary et al. found a significant deficiency of serum albumin in children with active CD using the PCDAI index which correlated with anti-Saccharomyces cerevisiae antibody titres [24].

The rates of serum vitamin deficiency were highest for vitamins A and E, principally in the patients with active CD (p < 0.05). This finding was consistent with Bousvaros et al. [25], who found that vitamin A deficiency and vitamin E deficiency were associated with disease activity. Hoffbrand et al. [26] found changes in plasma levels of antioxidant vitamins in CD patients in comparison with patients with ulcerative colitis and controls.

The limitations of the study included the lack of prealbumin; given its shorter half-life than that of albumin, it is a very good parameter to evaluate nutritional status.

## Conclusions

Adolescents with CD, including those with mildly to moderately active or inactive disease, are at risk for nutritional deficits, which makes it important to conduct nutritional assessments as part of the routine evaluation of such patients, in order to plan nutritional support as part of the overall treatment strategy in this population.

## Abbreviations

BMI: Body mass index
CD: Crohn’s disease
DRIs: Dietary reference intakes
IBD: Inflammatory bowel disease
